# Global Research Trends on Inflammation in Polycystic Ovary Syndrome: Insights From Bibliometric and Scientometric Analysis

**DOI:** 10.1155/ije/8861740

**Published:** 2025-10-30

**Authors:** Tingting Feng, Qin Wang, Fujin Shen, Jing Yang

**Affiliations:** ^1^Department of Obstetrics and Gynecology, Renmin Hospital of Wuhan University, Wuhan, Hubei, China; ^2^Reproductive Medicine Center, Renmin Hospital of Wuhan University, Wuhan, Hubei, China

**Keywords:** bibliometric analysis, CiteSpace, inflammation, polycystic ovary syndrome, VOSviewer

## Abstract

**Background:**

Polycystic ovary syndrome (PCOS) is a complex reproductive-endocrine disorder with an ambiguous etiology, presenting significant challenges for clinical management. Recently, inflammation has been identified as a pivotal factor in the pathogenesis of PCOS, attracting considerable attention within the scientific community. In this study, we used bibliometric and visualization analyses to investigate the research hotspots and developmental trends associated with inflammation in PCOS to obtain valuable insights for future research.

**Methods:**

The core collection of the Web of Science database was accessed to retrieve literature on inflammation and PCOS published from 2004 to 2024. Bibliometric analysis and visualization were performed using software tools including CiteSpace, VOSviewer, and GraphPad Prism. The analysis focused on publication year, country, institution, journal, author, publication, and literature keywords.

**Results:**

From January 1, 2004, to July 25, 2024, a total of 2568 articles were published on PCOS and inflammation, which involved 10,920 authors from 2902 research institutions across 92 countries. The volume of literature in this field had consistently increased, with significant contributions from the United States and China. Tehran University of Medical Sciences and the Egyptian Knowledge Base were identified as the most active and influential institutions. “Gynecological Endocrinology” was the leading journal in terms of the number of relevant articles published. Among the published authors, Asemi and Zatollah had the highest publication counts, while Azziz R.'s work received the most citations. Commonly occurring keywords included insulin resistance, obesity, and oxidative stress. Recent literature clustering and keyword analysis identified that resveratrol, granulosa cells, follicular fluid, gut microbiota, and probiotics are current research hotspots in this field. Gut microbiota and infertility are recognized as significant directions for future research.

**Conclusions:**

This bibliometric analysis provides a detailed overview of research on inflammation and PCOS, highlighting its importance in disease progression and the potential for therapeutic targeting of inflammatory pathways.

## 1. Introduction

Polycystic ovary syndrome (PCOS) is a prevalent endocrine disorder affecting approximately 5%–10% of women of reproductive age worldwide [1]. It is characterized by irregular menstruation, hyperandrogenemia, and/or polycystic ovarian morphology, with considerable variability in symptom presentation among individuals [2]. Beyond its impact on fertility, PCOS has significant long-term health consequences, including type 2 diabetes [3], cardiovascular disease [4], and endometrial cancer [5], which impose substantial economic and social burdens.

Recent research reports that PCOS patients often exhibit chronic low-grade inflammation and they often exhibit elevated levels of inflammatory markers, such as white blood cells (WBCs), C-reactive protein (CRP), interleukin-8 (IL-8), interleukin-6 (IL-6), interleukin-18 (IL-18), and tumor necrosis factor-α (TNF-α) compared to healthy individuals [6, 7]. Approximately 50%–70% of PCOS patients also show varying degrees of insulin resistance (IR), which is more pronounced in those with higher inflammatory marker levels. These inflammatory factors exacerbate IR and disrupt glucose and lipid metabolism by altering the balance of reactive oxygen species (ROS). In PCOS patients with hyperandrogenism (HA), elevated testosterone levels may stimulate the production of pro-inflammatory factors through the activation of inflammatory signaling pathways such as NF-κB, creating a harmful feedback loop [8, 9]. Moreover, the presence of lymphocytes and macrophages in the ovarian tissue of PCOS patients is significantly greater than that of healthy individuals. These immune cells release inflammatory factors and activate NF-κB, potentially leading to early apoptosis of granulosa cells (GCs) and inhibiting the development of dominant follicles, ultimately affecting ovarian function [10]. Previous studies have shown that disruption of the gut microbiota may play a role in the pathogenesis of PCOS, including IR, chronic inflammation, and obesity [11, 12]. Therefore, the complex interplay between inflammation and PCOS necessitates further systematic investigation.

Bibliometrics is a multidisciplinary field that combines mathematics, statistics, and philology to quantitatively analyze knowledge carriers and explore the underlying knowledge structure and dynamic changes in scientific documents. This approach can be used to reveal trends, hotspots, and potential future directions in scientific research [13, 14]. Despite the growing body of research on the relationship between inflammation and PCOS, systematic and professional analyses remain limited. In this study, we used bibliometric methods to identify active countries and regions, key research institutions and scholars, and evolving research trends. The findings aim to provide valuable insights for designing and conducting future studies, enhancing our understanding on the complex relationship between inflammation and PCOS, and suggesting promising research directions for future investigative and therapeutic efforts.

## 2. Materials and Methods

### 2.1. Data Sources and Search Strategy

To ensure a comprehensive analysis, we collected relevant data from the Web of Science Core Collection (WoSSC), one of the world's largest electronic scientific literature databases. The search strategy was formulated as follows: ((((((((((((TS = (“Polycystic Ovary Syndrome”)) OR TS = (“Ovary Syndrome, Polycystic”)) OR TS = (“Syndrome, Polycystic Ovary”)) OR TS = (“Stein-Leventhal Syndrome”)) OR TS = (“Stein Leventhal Syndrome”)) OR TS = (“Syndrome, Stein-Leventhal”)) OR TS = (“Sclerocystic Ovarian Degeneration”)) OR TS = (“Ovarian Degeneration, Sclerocystic”)) OR TS = (“Sclerocystic Ovary Syndrome”)) OR TS = (“Polycystic Ovarian Syndrome”)) OR TS = (“Ovarian Syndrome, Polycystic”)) OR TS = (“Polycystic Ovary Syndrome”)) OR TS = (“Sclerocystic Ovaries”)) OR TS = (“Ovary, Sclerocystic”)) OR TS = (“Sclerocystic Ovary”)) OR TS = (PCOS) AND ((TS = (Inflammation)) OR TS = (Inflammations)) OR TS = (Inflammatory)).

### 2.2. Inclusion and Exclusion Criteria

**Figure 1:**
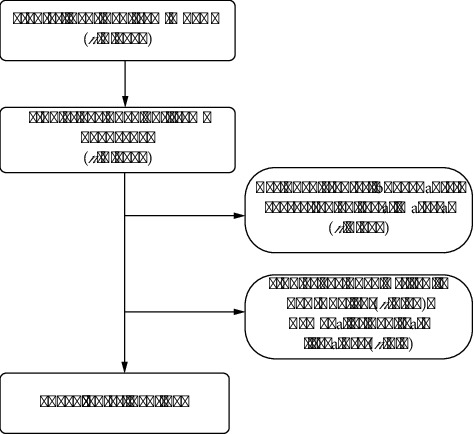
Flowchart showing the literature screening process.

Literature selection for this study was based on the following inclusion criteria: (1) full-text publications addressing inflammation in PCOS; (2) articles and reviews published in English; (3) publications dated from January 1, 2004, to July 25, 2024. The exclusion criteria were as follows: (1) topics unrelated to inflammation in PCOS; (2) conference abstracts, news items, briefings, and similar materials (Figure 1). Data selection was performed by two researchers (Tingting Feng and Qin Wang), who recorded and verified the content for thematic relevance. In cases of disagreement, a consensus was reached through mutual discussion or, if necessary, by consulting a third party for a final decision.

### 2.3. Statistical Analysis

We conducted bibliometric and knowledge mapping analyses on the collected data using CiteSpace (version 6.2.4R), VOSviewer (version 1.6.18), and GraphPad Prism (version 8.0.2). For CiteSpace analysis, the time slicing was set from January 2004 to July 2024 with 1-year intervals. The selection criteria were set to g-index (*k* = 25). Pathfinder and Pruning sliced networks algorithms were applied to simplify the network visualizations. To enhance the accuracy and reliability of our findings, we performed keyword co-occurrence analysis by consolidating synonyms, removing irrelevant terms, and standardizing the spelling of author and institution names. GraphPad Prism was used to generate linear graphs illustrating the number of publications and annual citation counts [15]. VOSviewer, a Java-based open-source software developed by van Eck and Waltman, is used to manage extensive bibliographic data and to determine relationships and domain structures in the scientific literature through co-citation and co-word analyses [16]. CiteSpace, designed by Professor Chen, is used to analyze scientific literature by incorporating visualization methods and showcasing the structure and dynamic evolution of scientific knowledge [17]. Both VOSviewer and CiteSpace are essential for visualizing and interpreting fundamental patterns within scholarly communication networks.

## 3. Results

### 3.1. Annual Publication Growth Trend

**Figure 2:**
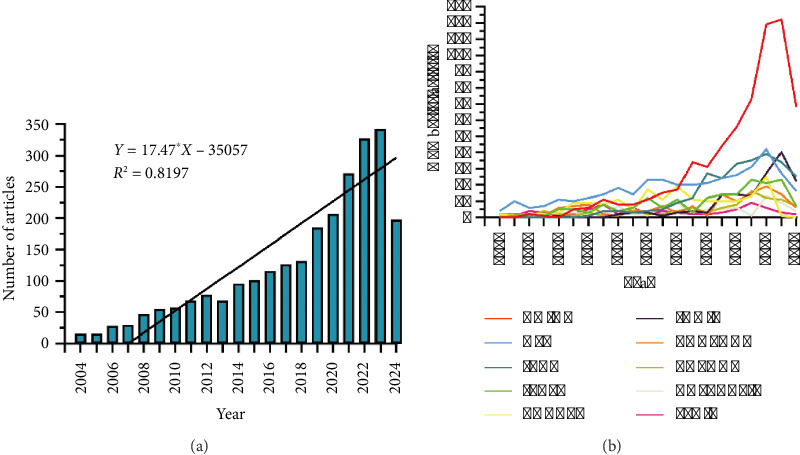
(a) Annual number of publications. (b) Line graph showing the trend of national publications over time.

From January 1, 2004, to July 25, 2024, a total of 2568 publications related to PCOS inflammation were identified in the WoSCC database. This corpus included 1870 articles and 698 reviews, contributed by 93 countries and regions, 2902 institutions, and 10,920 authors. The publication trend can be divided into three distinct phases: (1) early stage (2004–2007): During this period, the number of published articles increased slowly, with fewer than 30 articles per year. This relatively low output may reflect the limited attention the field received from the academic community at the time, potentially due to the complexity of PCOS and insufficient understanding of the role of inflammatory mechanisms in the disease. (2) Rapid growth stage (2008–2018): The number of publications increased significantly, indicating rapid development in the field. This growth may be attributed to advancements in molecular biology and immunology, which enabled a deeper exploration of the role of inflammation in PCOS. (3) Current stage (2019–2024): Since 2019, the number of publications has further increased, reaching its peak in 2023. This increase highlights the growing global attention to women's health issues and the expanding research interest in PCOS (Figure 2(a)).

### 3.2. Analysis of Countries/Regions

**Figure 3:**
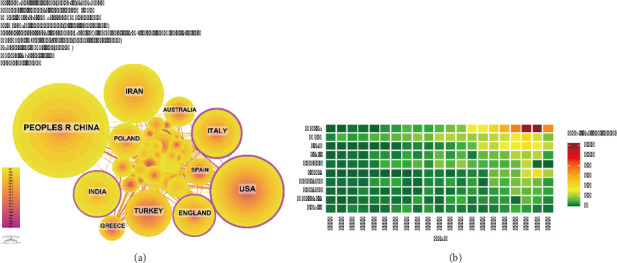
(a) A map illustrating the network of international collaborations. (b) Heat map representing the distribution of national publications.

Over the past two decades, research on PCOS and inflammation has involved 92 countries and regions. As detailed in Table 1, the leading countries in this field were China, the United States, Iran, Italy, and Turkey. China, initially having a low publication output, saw a rapid increase after 2016 (Figures 2(b), 3(b)).

Among the top 10 countries and regions, the United States stood out with 22,380 citations, significantly surpassing other nations. The average citation count for papers published in the United States was 59.05, the highest among the top contributors. This substantial figure underscored the United States' significant influence and recognition in the field of PCOS and inflammation research, reflecting the high overall quality of its research outputs. China, which has published a total of 630 articles, the highest among all countries, has received 10,732 citations, ranking second globally. Despite this substantial publication output, the average citation count for Chinese articles was 17.03, which was lower compared to that of other leading countries (Table 1).  Figure 3(a) illustrates the international collaboration network, highlighting that the United States maintained strong cooperative ties with countries such as the United Kingdom, Italy, and Spain. Conversely, China has established close research partnerships with Iran, Poland, India, and others, which plays an essential role in advancing research on PCOS and inflammation.

### 3.3. Analysis of Institutions

**Figure 4:**
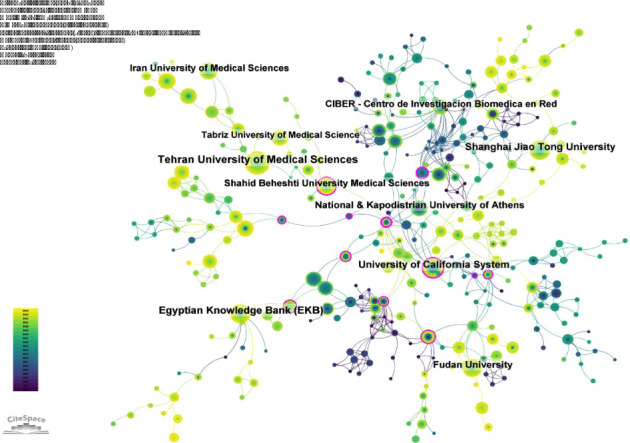
Institutional cooperation network illustrating the network of collaboration among institutions involved in the research on PCOS inflammation.

A total of 2902 institutions have systematically contributed to the literature on the role of inflammation in PCOS.  Table 2 provides a detailed list of the top 10 institutions based on publication volume. Notably, four of these institutions were from Iran, two from the United States, two from China, one from Greece, and one from Egypt (Table 2). Tehran University of Medical Sciences published the largest number of articles, 65 in total, with 1400 citations, and an average citation rate of 21.54. The Egyptian Knowledge Bank (EKB) ranked second with 51 articles, 571 citations, and an average citation rate of 11.20. The University of California followed in third place with 47 publications, 2680 citations, and an average citation rate of 57.02. Shanghai Jiao Tong University ranked fourth with 45 papers, 869 citations and an average citation rate of 19.31. Further analysis revealed that the degree of collaboration among these leading institutions was relatively low, suggesting that enhancing international cooperation and promoting broader academic exchanges could be pivotal in advancing research in this field (Figure 4).

### 3.4. Analysis of Journals

**Figure 5:**
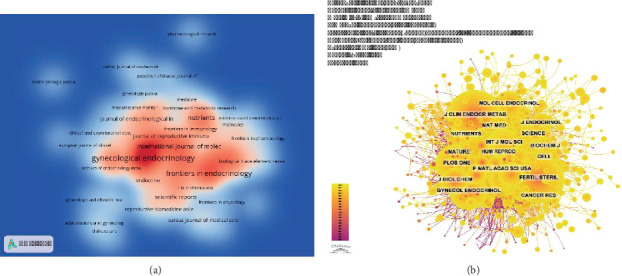
(a) Density map of journal publications. (b) Cocitation network map of journals.

To evaluate the prominence and influence of journals publishing research on PCOS and inflammation, a visual analysis of both published and cited journals was conducted. As shown in Table 3 and Figure 5(a), Gynecological Endocrinology was found to be the leading journal with the greatest number of articles, totaling 101 articles (3.93%), followed by Frontiers in Endocrinology (75 articles, 2.92%), Journal of Clinical Endocrinology & Metabolism (72 articles, 2.80%), Nutrients (49 articles, 1.91%), and International Journal of Molecular Sciences (47 articles, 1.83%). Among these, Fertility and Sterility had the highest impact factor of 6.6, followed by Human Reproduction with an impact factor of 6.0. Both journals are highly regarded and are classified in the Q1 or Q2 categories, reflecting their strong reputation and significant impact within the academic community.

The impact of a journal is frequently assessed using various metrics, one of which is co-citation frequency. As shown in Table 4, the Journal of Clinical Endocrinology & Metabolism was found to be the most cited with 2034 citations, followed by Fertility and Sterility with 1829 citations and Human Reproduction with 1572 citations. Interestingly, Endocrine Reviews, despite having been cited 908 times, had the highest impact factor (IF) of 22.0 among the top 10 journals. These co-cited journals predominantly are located in Quartile 1 (Q1) or Q2 categories and have been found to have mutual citation relationships, primarily within the fields of endocrinology and reproductive science (Figure 5(b)).

**Figure 6:**
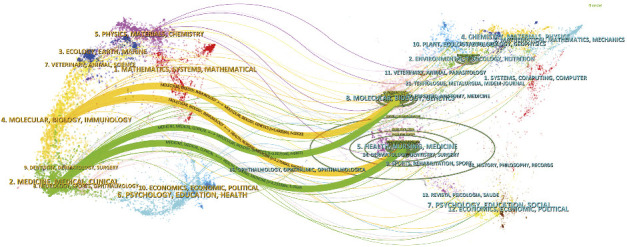
Dual map overlay of journals in this research field.

Figure 6 illustrates a dual-map overlay analysis of journals, which visually represented the flow of knowledge and research trends across different academic disciplines. This analysis identified key citation pathways, demonstrating that research published in the fields of Molecular/Biology/Genetics and Health/Nursing/Medicine was often cited by journals in Molecular/Biology/Immunology and Medicine/Medical/Clinical domains. The color-coded trajectories in the overlay provide a visual representation of the interconnectedness of these disciplines and their collective impact on the advancement of scientific knowledge.

### 3.5. Analysis of Authors

**Figure 7:**
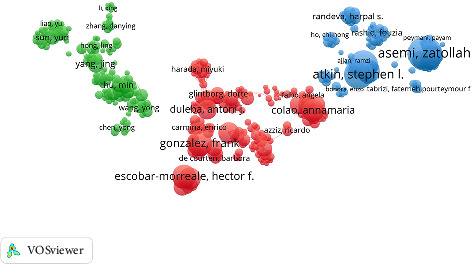
Co-occurrence network of authors.

The research literature on PCOS and inflammation comprised contributions from a total of 10,920 authors. Among them, approximately 190 authors have published five or more papers. As shown in Table 5, the top 10 authors collectively contributed 240 articles, which accounted for 9.35% of the total publications in this field. Zatollah Asemi from Kashan University of Medical Sciences and Health Services led this group with 39 articles. He was closely followed by Stephen L. Atkin from the Royal College of Surgeons in Ireland and Thozhukat Sathyapalan from the University of Hull, each with 30 articles. Additionally, Frank Gonzalez has published 24 articles, and Hector F. Escobar-Morreale has published 22 articles. The collaboration network among these authors is shown in Figure 7, highlighting key individuals, collaborative groups, and emerging research trends.

**Figure 8:**
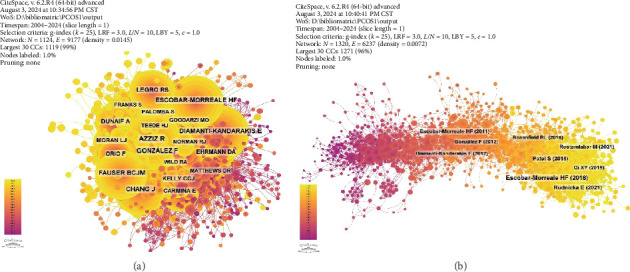
(a) Cocitation network of authors. (b) Cocitation network of the literature.

Cocitation analysis, which tracks instances where two or more authors are cited together in subsequent papers, provides insights into academic relationships and collaborations. This analysis is valuable for understanding the background and developmental trends within the research field. According to Table 5, 146 authors have been cited more than 50 times, indicating their significant influence and prominence in the discipline. The most frequently cited author was Azziz R., with 675 citations, followed by Diamanti-Kandarakis E. with 646 citations, Escobar-Morreale H. F. with 585 citations, Fauser B. C. with 527 citations, and Gonzalez F. with 515 citations. The level of collaboration among these authors varies, reflecting a range of research networks and collaborative dynamics (Figure 8(a)).

### 3.6. Analysis of Cited and Cocited References

Visual literature analysis can provide an intuitive understanding of the research focus, current trends, and future directions for PCOS-related inflammation. By selecting “references” as the node type, setting the publication year range from 2004 to 2024, and generating a connected cocitation network over one year, we obtained a comprehensive view of the field.

The resulting network consisted of 1320 nodes interconnected by 6237 links, as illustrated in Figure 8(b).  Table 6 lists the 10 most cited articles, which focused on the impact of inflammation on PCOS, potential treatment approaches, and new perspectives on its etiology. The most cited article, authored by Escobar-Morreale H. F. et al. from the University of Barcelona and published in Nature Reviews Endocrinology, has received 161 citations, reflecting the journal's high impact factor of 31.0. The next two most cited articles were published in the International Journal of Molecular Sciences and the Journal of Steroid Biochemistry and Molecular Biology, with 92 and 81 citations, respectively. These articles highlighted the significant relationship between inflammation and PCOS. In addition, the fourth-ranked study by Qi Xiaoxia, published in Nature Medicine in 2019, showed that an imbalance in the gut microbiota was also a factor associated with the occurrence of PCOS.

**Figure 9:**
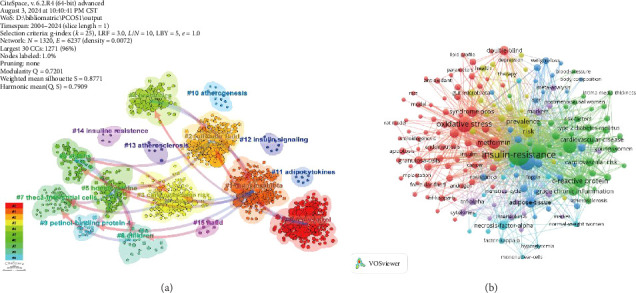
(a) Clustering map of cocited literature. (b) Network map of high-frequency keywords.

**Figure 10:**
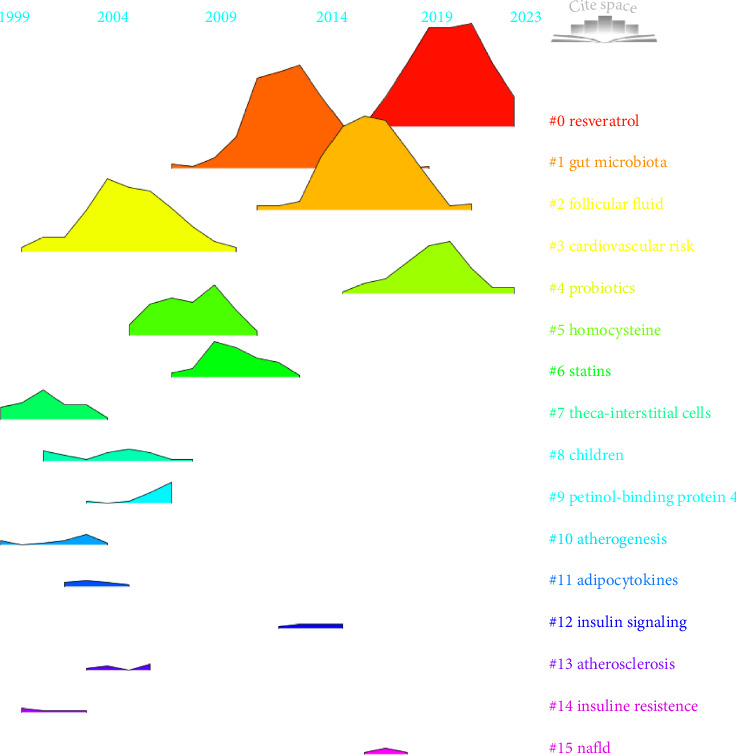
Peak map of cocited literature clustering.

A cluster analysis of co-cited references and time series data revealed the evolution and current focus of research in this field (Figures 9(a) and 10). Early research concentrated on topics such as cardiovascular risk (cluster 3), theca-interstitial cells (cluster 7), children (cluster 8), retinol-binding protein 4 (cluster 9), atherogenesis (cluster 10), adipocytokines (cluster 11), atherosclerosis (cluster 13), and IR (cluster 14). Mid-term research hotspots included homocysteine (cluster 5), statins (cluster 6), and insulin signaling (cluster 12). Recent trends have shifted towards areas such as resveratrol (cluster 0), gut microbiota (cluster 1), follicular fluid (cluster 2), probiotics (cluster 4), and nonalcoholic fatty liver disease (cluster 15). Notably, gut microbiota (cluster 1) has connections with multiple themes and can exist independently, while resveratrol (cluster 0) is linked to clusters 2 and 3 (Figure 9(a)).

**Figure 11:**
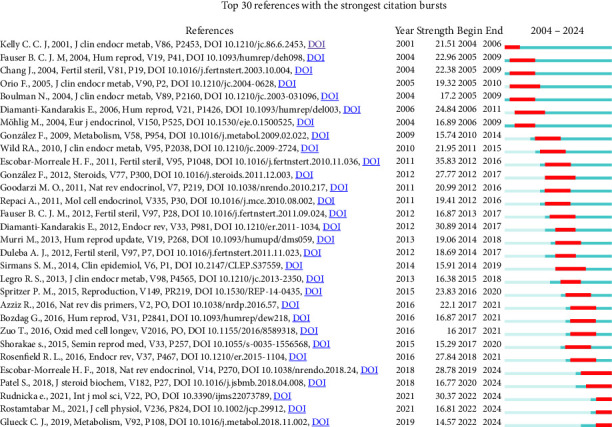
Bursting map of cited literature.

Through citation burst analysis, we identified the 30 papers with the most significant citation bursts (Figure 11). The article titled “Circulating inflammatory markers in PCOS: a systematic review and meta-analysis,” authored by Héctor F. Escobar-Morreale et al. and published in Fertility and Sterility, demonstrated the highest burst intensity, reaching 35.83. This study employed a systematic review and meta-analysis to comprehensively assess the levels of inflammatory markers in the blood of patients with PCOS, highlighting the important role of chronic low-grade inflammation in the pathogenesis of PCOS. In addition, a recent high-impact paper by Rudnicka E., with the burst intensity of 30.37, explored the relationship between inflammation and PCOS, revealing that a persistent inflammatory state was associated with multiple adverse clinical outcomes in PCOS patients. Furthermore, chronic inflammation during pregnancy was linked to an increased risk of complications such as miscarriage and placental dysfunction. Notably, this paper was also among the most cited in the field (Table 6). Currently, five articles continue to exhibit strong citation bursts, indicating ongoing research interest and impact in these areas.

### 3.7. Analysis of Keywords

**Figure 12:**
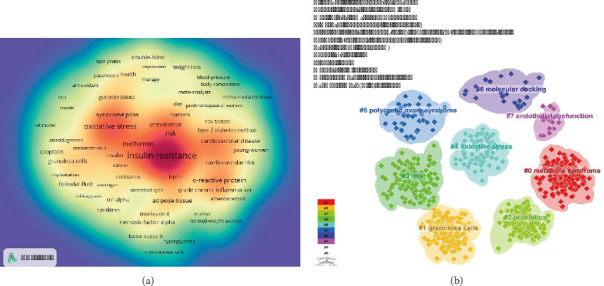
(a) Density map of keywords. (b) Clustering map of keywords.

Keyword indexing is an effective method for capturing the core content of scientific research papers and identifying emerging trends and hot topics. Based on the co-occurrence of keywords analyzed using VOSviewer, “insulin resistance” was the most frequently occurring term, with 969 mentions, followed by “obesity” with 526 mentions, “oxidative stress” with 457 mentions, “metabolic syndrome” with 313 mentions, and “C-reactive protein” with 272 mentions (Table 7, Figures 9(b), 12(a)). After removing irrelevant keywords, we identified a network of 180 keywords, each appearing at least 23 times, which formed 6 unique clusters. The largest cluster, marked in red, contained 70 keywords related to oxidative stress, metabolism, apoptosis, biomarkers, diagnostics, reproductive health, and vitamin D. This cluster likely focused on the pathophysiological mechanisms of PCOS, encompassing metabolic disorders, inflammation, and cell death. Cluster 2 (green) included 24 keywords, such as IR, metabolic syndrome, diabetes, and cardiovascular risk, indicating a focus on metabolic diseases and cardiovascular complications associated with PCOS, as well as inflammatory markers and endothelial function. Cluster 3 (blue) consisted of 26 keywords, including obesity, adipose tissue, and insulin sensitivity, suggesting an emphasis on the relationship between obesity and PCOS, particularly how obesity affects metabolism and inflammation. Cluster 4 (yellow) contained 22 keywords, such as androgen excess, consensus, depression, and endocrine characteristics, pointing to research on diagnostic criteria, endocrine characteristics, mental health status, and quality of life related to PCOS. Cluster 5 (purple) included 15 keywords, including chronic inflammation, cytokines, and inflammatory markers, indicating a focus on the role of inflammation in PCOS and associated genetic polymorphisms. Finally, Cluster 6 (sky blue) comprised 13 keywords, such as nuclear factor kappa B, HA, fatty acids, and inflammatory response, which may explore the specific mechanisms of the inflammatory response in PCOS, including the roles of immune cells and metabolic abnormalities.

**Figure 13:**
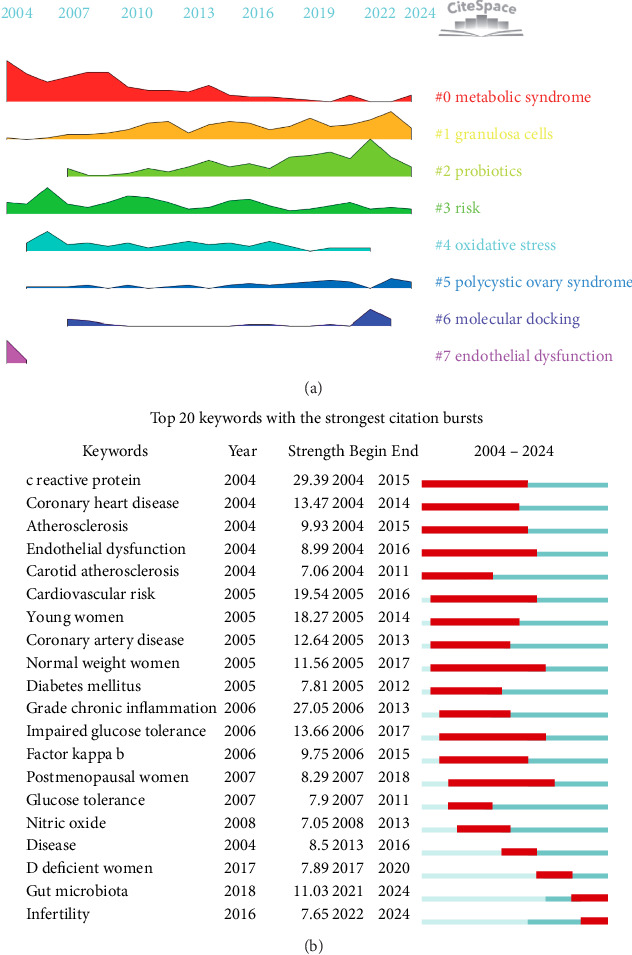
(a) Peak map of keyword clustering. (b) Bursting map of keywords.

To further investigate keyword clustering and its temporal trends, we performed a detailed analysis using CiteSpace software (Figures 12(b) and 13(a)). This analysis visually represents the development of research hotspots over time. Our findings suggested that topics such as “metabolic syndrome,” “granulosa cells,” “probiotics,” “risk,” “oxidative stress,” “polycystic ovary syndrome,” and “molecular docking” consistently ranked among the top subjects in this field. Notably, research on “granulosa cells” and “probiotics” has shown robust growth and widespread academic interest.

Furthermore, keyword burst detection has proven to be an effective method for rapidly identifying emerging research hotspots and developmental trends in research fields. As illustrated in Figure 13(b), we focused on the top 20 keywords with the most significant burst intensities. Among these, “C-reactive protein” displayed the highest burst intensity, with a value of 29.39 and a duration of 12 years since 2004, followed by “chronic inflammation,” which had a burst intensity of 27.05. Additionally, “gut microbiota” and “infertility” are currently in the outbreak stage, suggesting that they may become prominent research hotspots and promising directions for future studies.

## 4. Discussion

### 4.1. General Information

The increasing recognition of inflammation's role in PCOS has stimulated significant research in recent years. Since 2004, studies on the relationship between PCOS and inflammation have shown a steady growth trend, reflecting a growing global understanding of PCOS pathophysiology and the importance of inflammation in this condition. The United States and China have emerged as leading contributors to this field, with notable research activity also observed in countries such as Iran, Italy, and Turkey. This heightened activity in these countries may be attributed to their focus on women's health issues and substantial investments in scientific research.

Notably, a significant disparity in average citation per publication was observed between the United States (59.05) and China (17.03), despite China leading in total publication volume (Table 1). This discrepancy may be attributed to several factors. Firstly, researchers from the United States and other Western countries may have an advantage in disseminating their findings through high-impact international journals and conferences, leading to greater global visibility and earlier citation. Secondly, the research evaluation systems in different regions may prioritize different metrics; while some systems emphasize quantity and rapid output, others may place greater value on long-term, foundational, or clinically translational research that accumulates citations over a longer period. Thirdly, language barriers and regional research focus might limit the immediate international reach of some studies published by Chinese teams. Lastly, the stronger international collaboration network of the United States, as illustrated in Figure 3(a), likely facilitates more cross-border citation and academic influence.

In this field, the Tehran University of Medical Sciences and the EKB were at the forefront of research on the relationship between PCOS and inflammation, benefiting from long-term research efforts and dedicated teams. Similarly, Shanghai Jiao Tong University and Fudan University in China have also made significant contributions, publishing a substantial number of articles. Interestingly, four of the top 10 contributing institutions were from Iran, and together, they have published 167 articles. This high level of productivity can be linked to a thorough understanding of PCOS, effective resource management, and strong interdisciplinary collaboration.

Research on PCOS and inflammation has been predominantly published in high-impact endocrinology and reproductive journals, particularly those classified in the Q1 and Q2 categories. Journals such as “Gynecological Endocrinology” has a significant number of publications, “Fertility and Sterility” has the highest impact factor, and the “Journal of Clinical Endocrinology & Metabolism” has the highest citation counts. These journals exhibit mutual citation relationships, indicating a strong interconnectedness in the research community. The dual-map overlay analysis highlights that current research is largely focused on basic and clinical medicine, suggesting a need for expanded exploration to further advance the field. Zatollah Asemi from Iran has published the highest number of articles on the topic of PCOS. In addition, Azziz R., a distinguished scientist from the United States, ranked first among co-cited authors. Azziz R. has contributed extensively to the literature on PCOS, with significant publications covering a broad range of key topics. These include epidemiology, pathogenesis, and pathophysiology, as well as the various manifestations of PCOS, diagnostic criteria, screening and preventive measures, treatment and management strategies, long-term health effects, and future research directions [18, 19]. Diamanti-Kandarakis E. and Escobar-Morreale H. F. are also recognized as prolific and highly cited scholars in this field. Both researchers are esteemed experts in reproductive endocrinology and have made substantial contributions to PCOS research. They are notably involved in the development of important clinical guidelines and consensus statements, further advancing the understanding and management of PCOS [20–22].

The 10 most cited articles primarily investigated the role of inflammation in the pathogenesis of PCOS through various mechanisms, including insulin sensitivity, sex hormone metabolism, and the local ovarian microenvironment. The most cited article, authored by Escobar-Morreale H. F. et al. in 2018, provided a comprehensive review of the current understanding of PCOS. This article not only outlined existing gaps in knowledge but also offered evidence-based guidance for diagnosis and long-term clinical management [23].

### 4.2. Current Hotspots and Trends

A comprehensive review of inflammation research related to PCOS was conducted through literature and keyword trend analyses, revealing a broad range of research topics within the field of PCOS, with recent interests increasingly focusing on resveratrol (Category 0), gut microbiota (Category 1), follicular fluid (Category 2), and probiotics (Category 4).

Keyword co-occurrence analysis highlighted that “insulin resistance,” “obesity,” “oxidative stress,” and “metabolic syndrome” were the most frequently used terms. Numerous studies have explored the relationships between these factors and PCOS, underscoring their significant roles in the disease's pathophysiology [24–26]. Cluster analysis of keywords and their temporal correlations indicated that topics such as “metabolic syndrome,” “granulosa cells,” “probiotics,” “risk,” “oxidative stress,” “polycystic ovary syndrome,” and “molecular docking” have consistently garnered interest. Notably, “granulosa cells” and “probiotics” have emerged as prominent research hotspots. Keyword bursts indicated that “gut microbiota” and “infertility” are currently in the outbreak stage. The following sections will discuss the current research status on GCs, follicular fluid, gut microbiota, probiotics, resveratrol, and infertility in the context of PCOS and suggest potential future research directions.

#### 4.2.1. GCs and Follicular Fluid

GCs play a crucial role in the development, ovulation, and fertilization of oocytes by closely interacting with them [27, 28]. In individuals with PCOS, dysfunction of GCs is often considered to be one of the key factors in impaired follicle development and ovulation. Inflammation mediates tissue responses through complex immune pathways and plays an important role in biological processes. It is essential for physiological functions such as follicle formation and ovulation; however, ongoing chronic inflammation can hinder follicle growth and negatively impact subsequent reproductive potential [29]. A recent study found that overexpression of C1QTNF6 enhances the levels of inflammatory markers, including TNF-α, IL-6, and CRP in human ovarian GCs. This process activates the AKT/NF-κB pathway and contributes to the pathogenesis of PCOS [30]. Additionally, Xie et al. reported that inflammation and oxidative stress are present in the GC microenvironment of PCOS patients. Elevated expression levels of TNFα, IL-6, HIF-1*α*, and VEGFA were noted, which decreased GC proliferation and increased apoptosis. Treatment with chitosan oligosaccharide improved the inflammatory and oxidative stress conditions in these cells, promoting follicle development and endocrine function [31], which indicated that antioxidants or anti-inflammatory drugs may offer new therapeutic strategies for PCOS treatment.

Follicular fluid is a biological fluid found in growing secondary follicles and consists of plasma exudates rich in GC and follicular cell secretions. The relationship between the oocyte and surrounding GCs is closely connected through the follicular fluid microenvironment and provides a pathway for nutrient exchange and biological signal transmission. Therefore, the follicular fluid microenvironment plays a crucial role in the development and maturation of oocytes [32, 33]. Systematic analyses have revealed significant differences in follicular fluid composition between women with and without PCOS. This change is closely linked to the pathophysiological process of PCOS and may affect oocyte quality and reproductive outcomes during IVF [34]. A significant pro-inflammatory environment exists in the follicular fluid of PCOS [35–37]. After inflammatory factors in the peripheral circulation enter the follicles, they activate NF-κB through the IL-1 receptors and Toll-like receptor 4 on GCs and are transferred to the nucleus for activation. NF-κB can promote the gene expression of key components of the inflammasome NLRP3 and promote the cleavage of IL-1β and IL-18, thereby amplifying the inflammatory cascade and promoting GC apoptosis and ovarian fibrosis [38].

#### 4.2.2. Gut Microbiota and Probiotics

The gut microbiota is closely related to metabolic pathways, and disruption of the gut microbiota can lead to a variety of common metabolic diseases, including obesity, type 2 diabetes, nonalcoholic liver disease, metabolic heart disease, and malnutrition [39]. Research shows that changes in the gut microbiome are particularly common in PCOS [12, 40]. Sun et al. demonstrated that the gut microbiota composition in PCOS patients differs from that of healthy individuals, with reduced diversity and altered abundance of specific bacterial populations [41]. These changes can impair intestinal barrier function, trigger local inflammatory responses, and escalate to systemic inflammation [42]. PCOS patients are more prone to intestinal flora imbalance and abnormal microbiota, leading to the production of metabolic endotoxins. These endotoxins can penetrate the intestinal wall and increase inflammation throughout the body [43]. Studies have shown that endotoxemia may play an important role in chronic inflammation, IR, HA, and obesity in PCOS [44, 45]. Metabolites produced by the gut microbiota, such as bile acids, are also involved in the regulation of human metabolism. Following the transplantation of gut microbiota from PCOS patients into mice, as well as the administration of *Bacteroides vulgatus* to modify the gut microbiome of these mice, the subjects exhibited symptoms resembling those of PCOS, along with a reduction in the intestinal immune factor IL-22. Subsequent treatment of the mice displaying PCOS-like symptoms with glycocholic acid (GDCA) or IL-22 resulted in significant improvements in local ovarian inflammation, hormonal imbalances, irregular estrous cycles, polycystic ovary lesions, fertility challenges, and IR [46].

Modulating the gut microbiota can influence the metabolic state of the host, offering a novel strategy for treating diseases associated with metabolic disorders. Probiotics have the potential to enhance intestinal barrier function and mitigate inflammatory responses by regulating both the composition and function of intestinal flora [47]. Supplementation with probiotics has demonstrated potential in alleviating PCOS symptoms across various studies, positioning these interventions as viable strategies for managing the condition [48]. Recent meta-analyses have confirmed the beneficial effects of synbiotics and probiotics in regulating hormone levels and reducing inflammatory responses associated with PCOS [49]. Calcaterra et al. conducted a nonsystematic review, highlighting that probiotic and prebiotic supplements could significantly improve hormonal balance, metabolic function, and inflammatory markers in obese adolescents with PCOS. They also emphasized the importance of monitoring microbiota and administering probiotic supplements during childhood and early adolescence to prevent microbiota imbalances that might contribute to the onset and progression of PCOS [50].

In recent years, research on the gut microbiota has emerged as a rapidly advancing frontier in the field of PCOS. Therapeutic strategies targeting the gut microbiota—such as microbiota transplantation and probiotic interventions—have demonstrated considerable translational potential. Although animal studies remain valuable for untangling underlying mechanisms, the field is progressively transitioning toward clinical applications, further underscoring the clinical relevance of the gut microbiota as a therapeutic target for PCOS.

#### 4.2.3. Resveratrol

Resveratrol is a polyphenolic compound known for its diverse biological activities, which include anti-inflammatory [51], antioxidant [52], and antitumor effects [53]. Inflammation and oxidative stress are critical factors in the pathophysiology of PCOS. Research has demonstrated that resveratrol treatment can significantly alter the levels of inflammation-related cytokines and stress markers in the serum of PCOS patients, alleviating the pathological state of PCOS by inhibiting the inflammatory response and reducing endoplasmic reticulum stress [54]. Furthermore, studies indicate that resveratrol can attenuate the upregulation of TLR2 and its associated inflammatory responses in mice induced by a high-fat diet. It also inhibits the expression of TLR2 in LPS-stimulated GCs in a concentration-dependent manner, downregulating the mRNA and protein levels of pro-inflammatory mediators while reducing the expression of CAT, GPx, and SOD mRNA, thereby exerting antioxidative stress effects [55]. Resveratrol protects human GCs from oxidative stress and modulates inflammation and oxidative stress by activating the Nrf2 signaling pathway [56, 57]. Systematic reviews further indicate that resveratrol supplementation can effectively improve IR, dyslipidemia, ovarian morphology, and anthropometric indices in PCOS patients. Moreover, it may reduce inflammation and oxidative stress by regulating biological pathways, thereby alleviating PCOS symptoms [58].

#### 4.2.4. Infertility

Infertility is a major feature of PCOS, and PCOS patients often suffer from symptoms of metabolic disorders such as obesity, IR, and HA [29, 59, 60]. These metabolic disorders are closely related to inflammation. Abnormal inflammation can adversely affect the growth and development of follicles, inhibit ovulation, and lead to infertility [29, 61]. Furthermore, the metabolic disturbances associated with PCOS may exacerbate chronic inflammation, creating a vicious cycle that further impacts the patient's fertility. Treatment options for PCOS-related infertility include medication, surgery, and lifestyle interventions. These treatments can improve the endocrine and metabolic status of patients with PCOS and reduce the expression level of inflammatory factors to improve fertility. For instance, metformin can ameliorate chronic inflammation by enhancing metabolic parameters, lowering the production of inflammatory factors, and improving fertility outcomes [62]. Additionally, lifestyle changes, such as dietary management and moderate exercise, can effectively reduce weight and diminish inflammatory responses, thereby enhancing fertility in PCOS patients [63–65]. Nevertheless, infertility issues related to PCOS continue to be complex and challenging topics for future research.

## 5. Conclusion

Research on inflammation related to PCOS has progressed significantly, with substantial contributions from researchers in the United States and China. “Gynecological Endocrinology” has emerged as a leading journal in this domain. Notable scholars such as Zatollah Asemi and R. Azziz have made important contributions to the field. Current investigations focus on several key areas, including GCs, follicular fluid, gut microbiota, probiotics, resveratrol, and infertility. This present study provides an overview of current research trends, highlights emerging hotspots, and identifies limitations in the field, guiding future research directions and the potential impact of ongoing studies.

### 5.1. Limitations

This study has several limitations that should be clarified. Using solely the WoSCC database may have excluded relevant studies from other sources, such as Scopus or PubMed. While WoSCC is a leading database for bibliometric studies and provides a high-quality dataset, future research could benefit from a multi-database approach to further enhance comprehensiveness, acknowledging the potential variations in journal coverage between different platforms. Additionally, the quality of the included literature varies and might have affected the reliability of the conclusions drawn. The bibliometric tools used for data analysis also have inherent limitations. Furthermore, non-English and recently published studies might be underrepresented. Future research should address these limitations by incorporating a wider range of data sources and employing advanced analytical tools to achieve a more comprehensive understanding of PCOS and its inflammatory factors.

## Figures and Tables

**Table 1:** The top 10 productive countries by publication counts.

Rank	Country/region	Publication counts	Centrality	Percentage (%)	Citation	Citation per publication
1	China	630	0.03	24.53	10,732	17.03
2	USA	379	0.29	14.76	22,380	59.05
3	Iran	259	0.08	10.09	5550	21.43
4	Italy	181	0.21	7.05	6946	38.38
5	Turkey	179	0.02	6.97	3298	18.42
6	India	150	0.17	5.84	2134	14.23
7	England	135	0.16	5.26	4400	32.59
8	Poland	97	0.01	3.78	2289	23.60
9	Australia	86	0.01	3.35	4063	47.24
10	Spain	75	0.07	2.92	3175	42.33

**Table 2:** The top 10 productive research institutions based on publication counts.

Rank	Institution	Country	Number of studies	Total citations	Average citation
1	Tehran University of Medical Sciences	Iran	65	1400	21.54
2	Egyptian Knowledge Bank (EKB)	Egypt	51	571	11.2
3	University of California System	USA	47	2680	57.02
4	Shanghai Jiao Tong University	China	45	869	19.31
5	Fudan University	China	42	587	13.98
6	National & Kapodistrian University of Athens	Greece	39	2111	54.13
7	Iran University of Medical Sciences	Iran	37	1059	28.62
8	CIBER - Centro de Investigacion Biomedica en Red	USA	35	1326	37.89
9	Shahid Beheshti University Medical Sciences	Iran	34	592	17.41
10	Tabriz University of Medical Science	Iran	31	531	17.13

**Table 3:** The top 10 productive academic journals by publication counts.

Rank	Journal	Publications	Percentage (*n* = 2568) (%)	IF	Quartile in category
1	Gynecological Endocrinology	101	3.93	2	Q2
2	Frontiers In Endocrinology	75	2.92	3.9	Q2
3	Journal Of Clinical Endocrinology & Metabolism	72	2.80	5	Q1
4	Nutrients	49	1.91	4.8	Q1
5	International Journal of Molecular Sciences	47	1.83	4.9	Q1
6	Journal Of Ovarian Research	45	1.75	3.8	Q1
7	Fertility And Sterility	38	1.48	6.6	Q1
8	Clinical Endocrinology	34	1.32	3	Q2
9	Human Reproduction	33	1.29	6	Q1
10	Scientific Reports	29	1.13	3.8	Q1

**Table 4:** Top 10 journals by number of cocitations in the field.

Rank	Cited journal	Cocitation	IF	Quartile in category
1	J Clin Endocr Metab	2034	5.0	Q1
2	Fertil Steril	1829	6.6	Q1
3	Hum Reprod	1572	6.0	Q1
4	Plos One	1148	2.9	Q1
5	Clin Endocrinol	1067	3.0	Q2
6	Gynecol Endocrinol	1062	2.0	Q2
7	Hum Reprod Update	945	14.9	Q1
8	Endocr Rev	908	22.0	Q1
9	Diabetes	907	6.2	Q1
10	Endocrinology	865	3.8	Q2

**Table 5:** Top 10 authors by number of publications and cocited authors in the field.

Rank	Author	Publications	Rank	Cocited author	Citations
1	Asemi, Zatollah	39	1	Azziz, R.	675
2	Atkin, Stephen L.	30	2	Diamanti-Kandarakis, E.	646
3	Sathyapalan, Thozhukat	30	3	Escobar-Morreale, H. F.	585
4	Gonzalez, Frank	24	4	Fauser, C. M.	527
5	Escobar-Morreale, Hector F.	22	5	Gonzalez, F.	515
6	Butler, Alexandra E.	21	6	Chang, J.	499
7	Colao, Annamaria	20	7	Legro, R. S.	444
8	Luque-Ramirez, Manuel	19	8	Dunaif, A.	354
9	Diamanti-Kandarakis, E.	18	9	Carmina, E.	319
10	Jamilian, Mehri	17	10	Ehrmann, D. A.	295

**Table 6:** The top 10 co-cited references.

Rank	Author(s)	Year	Journal	Total citations	Title
1	Escobar-Morreale H. F.	2018	Nature Reviews Endocrinology	161	Polycystic ovary syndrome: definition, etiology, diagnosis, and treatment
2	Rudnicka E.	2021	International Journal of Molecular Sciences	92	Chronic Low-Grade Inflammation in the Pathogenesis of PCOS
3	Patel S.	2018	Journal of Steroid Biochemistry And Molecular Biology	81	Polycystic ovary syndrome (PCOS), an inflammatory, systemic, lifestyle endocrinopathy
4	Qi X. Y.	2019	Nature Medicine	73	The gut microbiota-bile acid-interleukin-22 axis orchestrates polycystic ovary syndrome
5	Escobar-Morreale H. F.	2011	Fertility And Sterility	68	Circulating inflammatory markers in polycystic ovary syndrome: a systematic review and meta-analysis
6	Rostamtabar M.	2021	Journal Of Cellular Physiology	66	Pathophysiological roles of chronic low-grade inflammation mediators in polycystic ovary syndrome
7	Rosenfield R. L.	2016	Endocrine Reviews	60	The Pathogenesis of Polycystic Ovary Syndrome (PCOS): The Hypothesis of PCOS as Functional Ovarian Hyperandrogenism Revisited
8	Gonzalez, Frank	2011	Steroids	56	Inflammation in Polycystic Ovary Syndrome: Underpinning of insulin resistance and ovarian dysfunction
9	Diamanti-Kandarakis E.	2012	Endocrine Reviews	56	Insulin Resistance and the Polycystic Ovary Syndrome Revisited: An Update on Mechanisms and Implications
10	Glueck C. J.	2019	Metabolism-Clinical And Experimental	55	Characteristics of obesity in polycystic ovary syndrome: Etiology, treatment, and genetics

**Table 7:** The most common keywords used.

Rank	Keyword	Counts
1	Insulin-resistance	969
2	Obesity	526
3	Oxidative stress	457
4	Metabolic syndrome	313
5	C-reactive protein	272
6	Prevalence	246
7	Metformin	231
8	Risk	228
9	Hyperandrogenism	198
10	Adipose-tissue	186
11	Syndrome PCOS	179
12	Grade chronic inflammation	141
13	Necrosis-factor-alpha	140
14	Double-blind	129
15	Cardiovascular-disease	128
16	Infertility	128
17	Cardiovascular risk	118
18	Granulosa-cells	102
19	Glucose	97
20	Metabolism	97

## Data Availability

The original contributions presented in the study are included in the article; further inquiries can be directed to the corresponding authors.
